# Transcriptome analysis reveals the genetic basis underlying the seasonal development of keratinized nuptial spines in *Leptobrachium boringii*

**DOI:** 10.1186/s12864-016-3295-9

**Published:** 2016-11-28

**Authors:** Wei Zhang, Yue Guo, Jun Li, Li Huang, Eric Gilbert Kazitsa, Hua Wu

**Affiliations:** Institute of Evolution and Ecology, International Research Centre of Ecology and Environment, School of Life Sciences, Central China Normal University, 152 Luoyulu, Hongshan District, Wuhan, 430079 China

**Keywords:** *Leptobrachium boringii*, Nuptial spine, Transcriptome, Estrogen, Insulin growth factor, Sexually selected traits

## Abstract

**Background:**

The expression of sexually selected traits often varies with populations’ breeding cycles in many animals. The elucidation of mechanisms underlying the expression of such traits is a research topic in evolutionary biology; however, the genetic basis of the seasonal development of their expression remains unknown. Male *Leptobrachium boringii* develop keratinized nuptial spines on their upper jaw during the breeding season that fall off when the breeding season ends. To illuminate the genetic basis for the expression of this trait and its seasonal development, we assessed the *de novo* transcriptome for *L. boringii* using brain, testis and upper jaw skin and compared gene expression profiles of these tissues between two critical periods of the spine growth cycle.

**Results:**

We identified 94,900 unigenes in our transcriptome. Among them, 2,131 genes were differentially expressed between the breeding period when the spines developed and the post-breeding period when the spines were sloughed. An increased number of differentially expressed genes (DEGs) were identified in the upper jaw skin compared with the testis and brain. In the upper jaw skin, DEGs were mainly enriched in cytosolic part, peptidase inhibitor activity and peptidase regulator activity based on GO enrichment analysis and in glycolysis/gluconeogenesis, ribosome biogenesis in eukaryotes and retinol metabolism based on KEGG enrichment analysis. In the other two tissues, DEGs were primarily involved in the cell cycle, DNA replication and melatonin production. Specifically, insulin/insulin-like growth factor and sex steroid hormone-related DEGs were identified in the upper jaw skin, indicating . The expression variation of IGF2 and estrogen-related genes may be the main factors regulating the seasonal development of the spines.

**Conclusions:**

Our study provides a list of potential genes involved in the regulation of seasonal development of nuptial spines in *L. boringii*. This is the first transcriptome survey of seasonally developed sexually selected traits for non-model amphibian species, and candidate genes provided here may provide valuable information for further studies of *L. boringii.*

**Electronic supplementary material:**

The online version of this article (doi:10.1186/s12864-016-3295-9) contains supplementary material, which is available to authorized users.

## Background

Elucidating the mechanisms underlying the expression of sexually selected traits (SSTs) has been an important research topic in evolutionary biology since Darwin’s theory of sexual selection was developed [1, 2]. Intrasexual selection (competition for mates) and intersexual selection (mate choice) are the two principle mechanisms driving the evolution of animal sexual traits [3]. In most species, males tend to compete with each other for acquiring mates, and females tend to be choosy. Thus, SSTs typically evolved in male individuals [1–3]. The best studied SSTs are elaborate ornaments or weapons, such as the peacock’s tail [4], horns of scarab beetles [5], swords of the swordtail fish [6], and antlers of the deer [7]. Among SSTs, weapons have evolved multiple times across the animal kingdom [8]. Specific to male individuals, weapons are often used in physical combat between male rivals competing for mates by establishing dominance hierarchies or defending reproduction resources [1, 3, 8, 9]. However, despite a wealth of information demonstrating the importance of weapons and their evolution by sexual selection, physiological and genetic mechanisms that regulate their development across animal taxa remain unknown [2, 8].

Within species, characteristics of male SSTs in general and the size of weapons in particular strongly depend on individual body size, physiological condition, nutritional condition and genetic quality [5, 10–14]. Moreover, the expression of diverse male SSTs varies with a populations’ breeding cycle [15–18]. Some male SSTs, such as ornamental plumage of birds, are highly expressed during the breeding season [19]. Other SSTs, such as the nuptial pads of amphibians, nuptial coloration of fishes, or nuptial spines of *Leptobrachium* anurans, only appear during the breeding season [15–18]. Seasonal changes in plasma sex steroid hormones, especially in testosterone, are important factors that regulate the growth of seasonally developed male sexual traits [16, 18, 20, 21]. The increase in testosterone (or related androgen) in males could trigger the expression of such traits, whereas low levels of this hormone would be insufficient to trigger its activation [18, 19, 22]. Recent studies have demonstrated that the insulin/insulin-like growth factor (IGF) pathway is a widely conserved physiological mechanism regulating the development of pronounced sexual traits across animal taxa [23, 24]. In addition, IGF can interact with sex steroid hormones to regulate the development of condition-sensitive sexual traits [25, 26]. However, prior research on the mechanisms controlling the expression of sexual traits mostly used physiological experiments, such as histology and hormone implants experiments, or measured expression levels of a small number of genes, making the genetic basis of these mechanisms largely unknown [15, 16, 20, 21, 27].

**Fig. 1:**
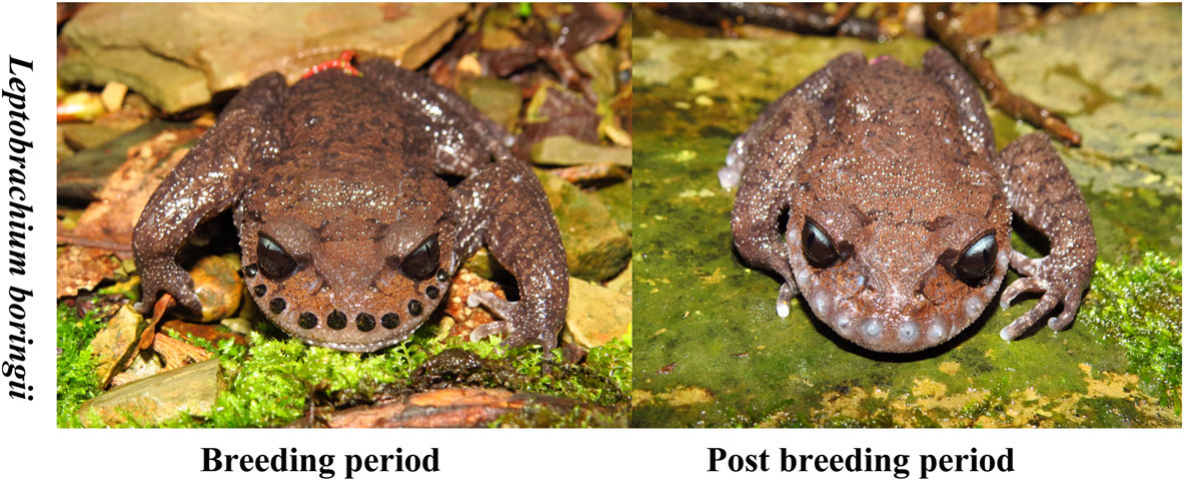
Different phenotypes of the keratinized nuptial spines in *Leptobrachium boringii* during different breeding periods. Male *L. boringii* grow 10 to 16 nuptial spines on the upper jaw during the breeding period (left), and the spines are sloughed at the post-breeding period (right)

In amphibians, most SSTs develop only during the breeding season [16]. In addition, hormonal-based modulatory mechanisms for the expression of some SSTs, such as skin excrescences at the thumb pads and nuptial pads, have been well studied in numerous anurans [16, 28]. Among amphibians, defensive structures such as spines and tusks are developed by males of few anuran species [29, 30]. They have been phenotypically described in diverse publications, but the physiological mechanisms regulating their development remain unexplored [16, 30]. In *Leptobrachium boringii*, during the breeding season, adult males develop 10 to 16 keratinized nuptial spines on the edge of their upper jaw that are used as weapons during combat with rivals for territory defense and acquiring mates, and these spines fall off once the breeding season ends (Fig. 1) [31, 32]. Therefore, *L. boringii* could be an excellent model to study the molecular basis underlying the seasonal development of sexually selected traits for seasonal breeding animals.

High-throughput next-generation sequencing technologies allow for the accurate exploration of gene expression profiles of phenotypic traits for living organisms. Differential gene expression analysis allows studying the genetic basis of context-specific tissue development without the need for a reference genome [33]. Here, we sequenced and assembled the transcriptome of the brain, testis and the upper jaw skin of male *L. boringii* during the breeding and post-breeding periods using Illumina sequencing technology. We then compared the differential gene expression profiles of the three tissues between these two time periods of the spine growth cycle. Our objective was to identify candidate genes involved in the seasonal development of nuptial spines in *Leptobrachium* anurans.

## Results and Discussion

### Illumina sequencing and *de novo* assembly

Due to the absence of a reference genome for *L. boringii*, we *de novo* assembled a transcriptome as a reference for read mapping and gene expression profiling in this species. The assembly generated 602,214,290 raw reads for *L. boringii.* We obtained an average of 31,817,564 clean reads for our 18 samples (Additional file 1: Table S1). In our assembly, 183,601 transcripts with a total length of 258,254,318 bp were obtained, and they were assembled into 94,900 unigenes, which totaled 81,414,717 bp. N50 lengths of the transcripts and unigenes were 2,941 bp and 1,854 bp, respectively (Table 1), and Q20 of all samples were greater than 96% (Additional file 1: Table S1). In addition, the mapping rates of clean reads to the assembled unigenes for all samples ranged from 75.70% to 81.83% (Additional file 1: Table S1). Taken together, our *de novo* assemblies revealed a high quality compared with previous studies [5, 34, 35]. This high quality of sequence reads and assembly was the foundation of all our subsequent analyses [36].

**Table 1:** Number and length of transcripts and unigenes in the *Leptobrachium boringii* transcriptome

Length	Transcripts	Unigenes
200–500 bp	79,093	58,375
500–1 k bp	33,563	16,031
1 k-2 k bp	29,559	9,898
>2 k bp	41,386	10,596
Total length	258,254,318	81,414,717
Mean length	1,407	858
N50	2,941	1,854
Total number	183,601	94,900

### Functional annotation and classification of unigenes

We used the databases NR, Swiss-Prot, PFAM, KOG, GO, and KEGG for unigene annotation. NR and Swiss-Prot databases resulted in reliable protein annotations for approximately 25% of unigenes. The number and percentage of annotated unigenes are presented in Table 2. Among all annotated databases, the largest match of annotation hits (24.51%) was obtained with the GO database. KEGG showed the smallest match (11.19%, Table 2). Of 94,900 unigenes assembled, 29,319 genes (30.89%) exhibited a positive match against at least one database. Thus, many of the unigenes have not been functionally annotated. However, several reasons could explain this annotation failure. Those unsuccessfully annotated unigenes could be partially or misassembled transcripts, sequences from UTR protein regions, or meaningless protein genes. In addition, for non-model amphibians, published genomic data in general and especially genetic data on the expression of sexual traits are still lacking. All of these factors combined with the absence of genomic information for *L. boringii* would have caused the failure of functional annotation of a part of the identified unigenes. However, considering that previous studies successfully annotated approximately 25,000 genes in other non-model amphibian species, such as *Odorrana margaretae*, *Megophrys sangzhiensis* and *Rhacophorus omeimontis* [34, 37], our gene annotation results (more than 29,000 unigenes annotated) from *L. boringii*’s transcriptome can be considered as high quality.

**Table 2:** Summary of unigenes annotated in different databases

Database	Annotated unigenes	Percentage (%)
NR	22,577	23.79
PFAM	22,121	23.30
Swiss-Prot	19,514	20.56
KOG	12,357	13.02
GO	23,260	24.51
KEGG	10,625	11.19
All	29,319	30.89

*NR* NCBI non-redundant protein sequences, *NT* NCBI non-redundant nucleotide sequences, *PFAM* Protein family, *Swiss-Prot* A manually annotated and reviewed protein sequence database, *KOG* Clusters of Orthologous Groups of proteins, *GO* Gene Ontology, *KEGG* Kyoto Encyclopedia of Genes and Genomes database, All, total number of unigenes that were successfully annotated in at least one database

**Fig. 2:**
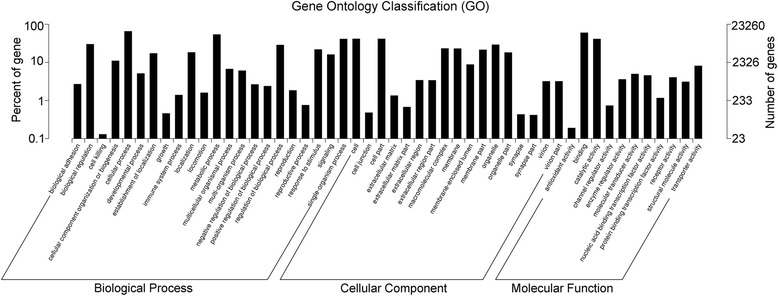
Gene Ontology (GO) classification of the assembled unigenes in *Leptobrachium boringii*

**Fig. 3:**
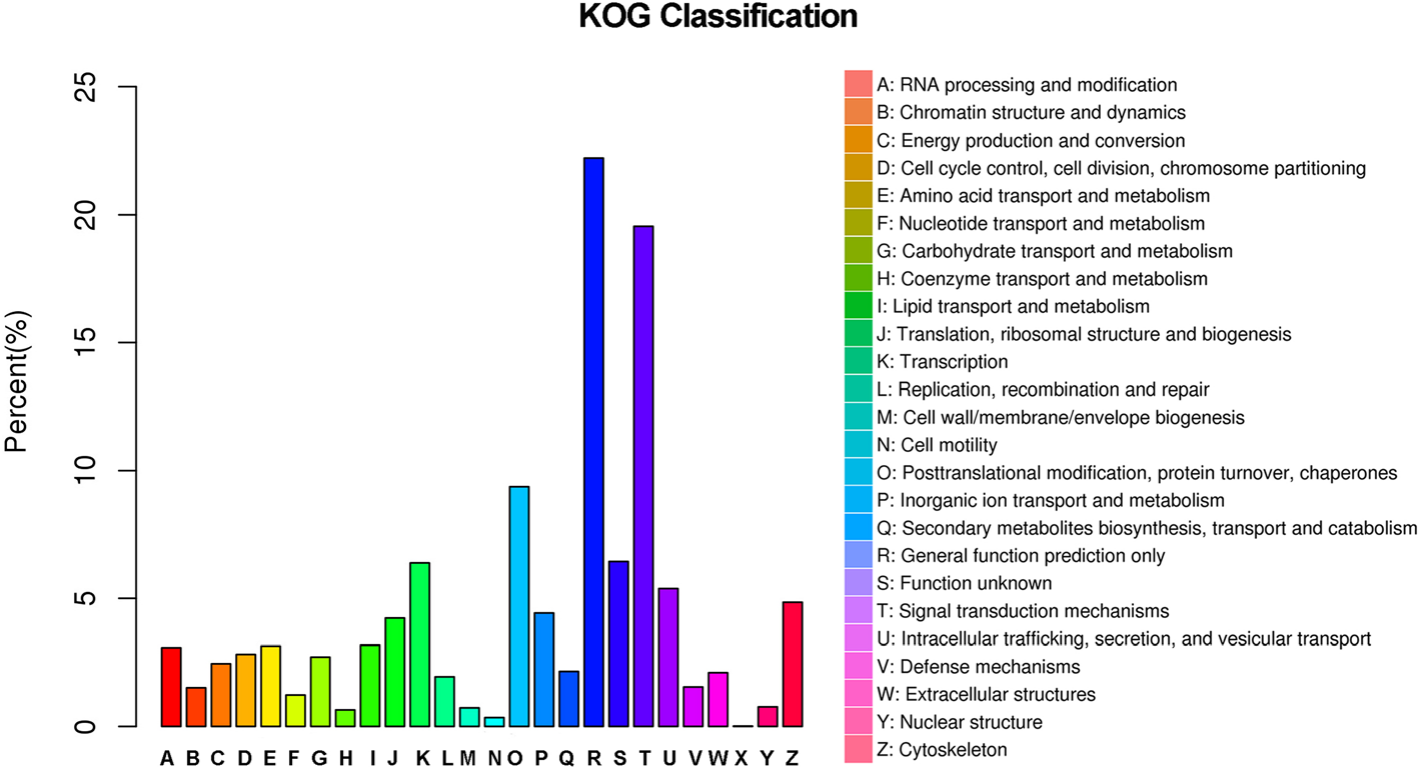
Eukaryotic orthologous group (KOG) classification of the assembled unigenes in *Leptobrachium boringii*

**Fig. 4:**
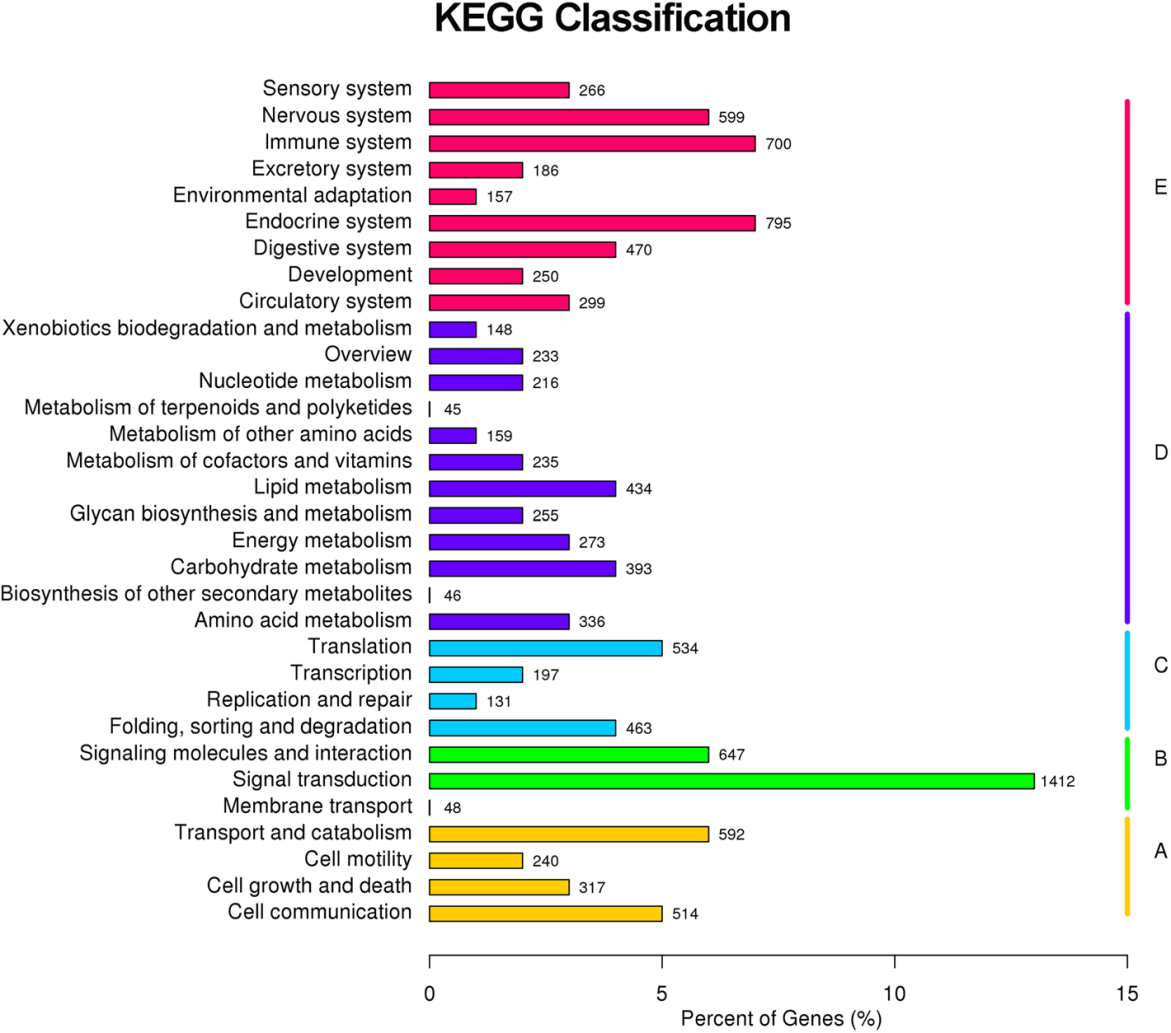
KEGG classification of the assembled unigenes in *Leptobrachium boringii*. A to E represent five categories of the KEGG pathways: **a** cellular processes; **b** environmental information processing; **c** genetic information processing; **d** metabolism; **e** organismal systems

In our study, a total of 23,260 unigenes (~25%) were assigned to 50 sub-categories of GO terms belonging to the following three main categories: cellular component (CC), molecular function (MF) and biological process (BP). These main categories included 17, 11 and 22 sub-categories, respectively (Fig. 2). The most enriched GO terms were related to cell and organelle parts for the category CC, binding and catalytic activity for MF, and cellular process and metabolic process for BP (Fig. 2). The KOG database mainly describes the phylogenetic classification of proteins encoded in complete genomes [38]. In our annotation, only a small proportion of assembled unigenes were mapped to the KOG database, with 12,357 genes classified functionally into 26 categories (Fig. 3). The most enriched KOG categories were General function prediction (2,746 or 22.2%), Signal transduction mechanisms (2,416 or 19.6%), and Posttranslational modification, protein turnover and chaperones (1,158 or 9.4%). In addition, we further annotated all unigenes into pathways in KEGG for understanding their high-level functions and utilities in the biological system of studied animals [39]. A total of 10,625 unigenes were mapped to 259 signaling pathways in our annotation. Signal transduction was the most enriched pathway, followed by pathways in the endocrine system and immune system (Fig. 4). These annotations may provide a valuable resource for further understanding of specific functions and pathways in *L. boringii* studies.

### Analysis of differentially expressed genes in the studied tissues

To explore genes controlling the seasonal development of nuptial spines in *L. boringii*, we compared the gene expression profiles in the brain, testis and upper jaw skin between the breeding and post-breeding periods and identified the significantly differentially expressed genes (DEGs). The final set of DEGs in each tissue was revealed by the hierarchical clustering of expression patterns (Additional file 2: Figure S1). The hierarchical clustering of DEGs revealed that sample groups were more divergent across tissues than between the studied time periods. Each tissue had a relatively distinct gene clusters and expression pattern, so we performed the following differential expression analysis separately for each studied tissue.

**Fig. 5:**
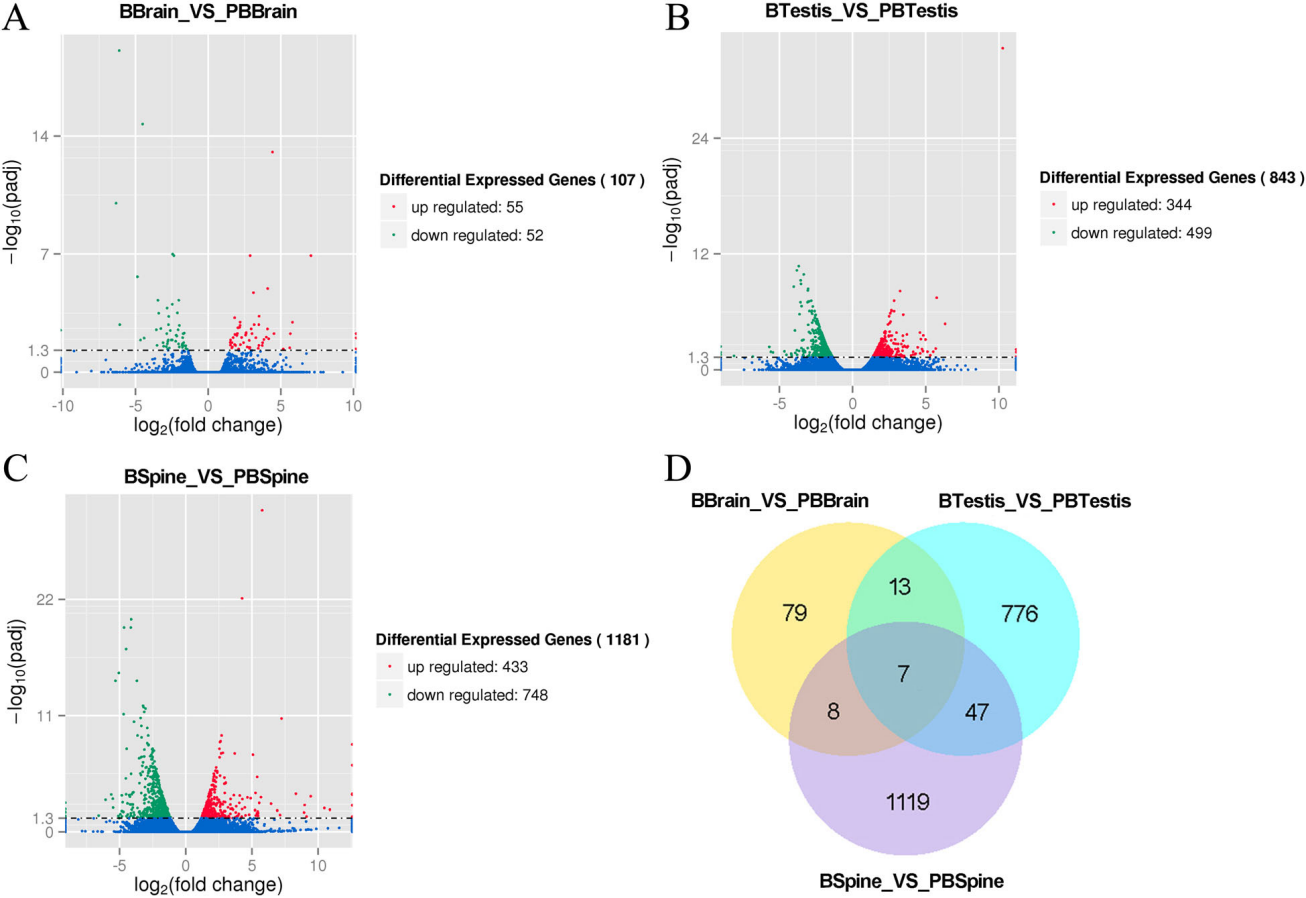
Differential expression analysis of tissue comparisons between the breeding and post-breeding periods. Volcano plot of differentially expressed genes in the brain (**a**), testis (**b**) and upper jaw skin (**c**; spine for short in figure) are separately presented. The X axis represents differential fold-change (log2FC) of the differentially expressed genes (DEGs), and the Y axis represents the –log padj value of the DEGs. padj represents the adjusted *P*-value of the DEG, and 0.05 was set as the significance level. Significantly up-regulated DEGs in the breeding period compared with the post-breeding period are presented in red, whereas significantly down-regulated DEGs are shown in green. Non-significantly differentially expressed genes are presented in blue. **d** Venn diagram showing co-differentially expressed genes among different tissue comparisons in *Leptobrachium boringii*

Overall, we finally identified 2,131 DEGs in the three tissues, and 75 DEGs were shared by these tissues (Fig. 5d). For approximately 70% (1,484) of the DEGs that were functionally annotated, we were able to complete an overall comprehensive review of these DEGs. As shown in the Venn diagram (Fig. 5d), more DEGs were found in the upper jaw skin compared with the brain and testis. This finding suggests that the upper jaw skin causes greater changes in gene expression than the sexual glands (testes) and central nervous system (brain) during different breeding periods in *L. boringii*, indicating that the upper jaw skin might play a relatively important role in the development of nuptial spines compared with the other two tissues. Significant GO enrichment and KEGG enrichment (*p* < 0.05) in each tissue could provide valuable information to understand the gene function of the DEGs involved in the development of nuptial spines (Table 3; Table 4; Additional file 1: Table S2).

**Table 3:** The most enriched GO terms of differentially expressed genes in the three tissues

Go term	*P*-value	DEG (all)	Representative genes
(a) Upper jaw skin: differentially expressed genes: 1181			
Cytosolic part (GO:0044445:CC)	<0.001	17 (148)	Rab interacting lysosomal protein; keratin, type I cytoskeletal 42-like; keratin 17; dystrophin related protein 2
Peptidase inhibitor activity (GO:0030414:MF)	<0.001	20 (232)	Collagen alpha-1(XXVIII) chain; Cathelicidin antimicrobial peptide; small nuclear ribonucleoprotein polypeptide A; serpin B5
Peptidase regulator activity GO:0061134:MF)	<0.001	20 (234)	Cystatin B (stefin B); tectorin alpha, gene 2 precursor; cystatin-like isoform 1; WAP four-disulfide core domain protein 3-like
Extracellular region part (GO:0044421:CC)	<0.001	49 (786)	Keratin 17; newt-specific cysteine-rich growth regulator; keratin, type II cytoskeletal 75-like; extracellular matrix protein 1 isoform 1
Prefoldin complex (GO:0016272:CC)	<0.001	11 (71)	Keratin, type I cytoskeletal 42-like; keratin 17;keratin, type I cytoskeletal 18-B; dystrophin related protein 2
Extracellular region (GO:0005576:CC)	<0.001	95 (1993)	Keratin 17; insulin-like growth factor-binding protein 2 precursor; transmembrane protein 111
(b) Testis: differentially expressed genes: 843			
Chromosome (GO:0005694:CC)	<0.001	51 (627)	Origin recognition complex, subunit 1; shugoshin-like protein 2; ubiquitin-conjugating enzyme E2 T
Chromosomal part (GO:0044427:CC)	<0.001	45 (538)	Tryptophan rich basic protein precursor; centromere protein I; proliferating cell nuclear antigen
Chromatin (GO:0000785:CC)	<0.001	26 (194)	DNA replication licensing factor mcm4; GTP-binding nuclear protein Ran; transcriptional activator Myb isoform 2
Cell cycle (GO:0007049:BP)	<0.001	50 (711)	Meiotic nuclear divisions 1 homolog; proliferating cell nuclear antigen; cyclin-dependent kinases regulatory subunit 2
MCM complex (GO:0042555:CC)	<0.001	6 (10)	DNA replication licensing factor mcm5; DNA replication licensing factor mcm4; zygotic DNA replication licensing factor mcm6
Chromatin binding (GO:0003682:MF)	<0.001	17 (131)	High mobility group nucleosomal binding domain 3; DNA replication licensing factor mcm4; transcriptional activator Myb isoform 2
(c) Brain: differentially expressed genes: 107			
Aralkylamine N-acetyltransferase activity (GO:0004059:MF)	0.002	1 (1)	Arylalkylamine N-acetyltransferase;
Selenium binding (GO:0008430:MF)	0.003	2 (41)	Selenoprotein P, plasma, 1-like precursor;
Sulfonylurea receptor activity (GO:0008281:MF)	0.004	1(2)	ATP-binding cassette sub-family C member 9 isoform 1;
Actin crosslink formation (GO:0007049:BP)	0.009	1 (5)	Actinin, alpha 3
Cysteine-type peptidase activity (GO:0008234:MF)	0.009	3 (241)	Cthepsin K-like; cathepsin L1 precursor;
Protein-hormone receptor activity (GO:0016500:MF)	0.014	1 (8)	Thyrotropin receptor isoform 1 precursor

The significantly enriched GO terms in each tissue of *Leptobrachium boringii* are presented above. For each GO term, its ID, the ontology of level 1 (BP, Biological Process; MF, Molecular Function; CC, Cellular Component), count of the number of differentially expressed genes (DEGs) and the number of all annotated genes are presented (All in brackets). The representative genes of each enriched GO term are also provided here

**Table 4:** Significantly enriched KEGG pathways among differentially expressed genes in *Leptobrachium boringii*

Tissue	DEGs type	Pathway	FDR	DEGs	All genes
Spine	All	Glycolysis/Gluconeogenesis	1.65E-02	14	87
	Up-regulated	Ribosome biogenesis in eukaryotes	7.88E-04	10	70
		Biosynthesis of secondary metabolites	3.12E-03	22	360
		Proteasome	1.68E-02	6	40
		Sulfur relay system	3.97E-02	3	9
		Pentose phosphate pathway	4.08E-02	5	35
		Aminoacyl-tRNA biosynthesis	4.40E-02	5	37
	Down-regulated	Retinol metabolism	8.78E-04	12	91
		ECM-receptor interaction	4.19E-03	11	98
		Tyrosine metabolism	4.19E-03	8	53
		Drug metabolism - cytochrome P450	1.20E-02	10	98
		Glycolysis / Gluconeogenesis	1.52E-02	9	87
		Nicotinate and nicotinamide metabolism	1.52E-02	6	39
		Vitamin B6 metabolism	2.30E-02	3	8
		Pantothenate and CoA biosynthesis	2.39E-02	4	18
Testis	All	Cell cycle	3.29E-11	27	125
		DNA replication	9.73E-11	14	30
		Cell cycle - yeast	1.38E-10	20	73
		Meiosis - yeast	4.89E-05	12	56
		Mismatch repair	5.80E-05	7	17
		Pyrimidine metabolism	5.76E-04	14	97
		Fanconi anemia pathway	3.52E-03	8	41
		Homologous recombination	5.07E-03	6	24
		Purine metabolism	1.78E-02	17	187
	Down-regulated	Cell cycle	0.00E + 00	26	125
		DNA replication	1.67E-13	14	30
		Cell cycle - yeast	3.46E-13	19	73
		Meiosis - yeast	3.19E-07	12	56
		Mismatch repair	2.53E-06	7	17
		Pyrimidine metabolism	1.11E-04	12	97
		Fanconi anemia pathway	1.29E-04	8	41
		Homologous recombination	3.89E-04	6	24
		Base excision repair	1.57E-02	5	31
		Nucleotide excision repair	2.18E-02	5	34
		Spliceosome	2.31E-02	10	133
		Oocyte meiosis	2.31E-02	9	112
		Purine metabolism	2.74E-02	12	187
		p53 signaling pathway	4.31E-02	6	61

The significantly enriched KEGG pathways in each tissue of *Leptobrachium boringii* are presented above. FDR represent the false discovery rate adjusted *P*-value. For each enriched KEGG pathway, we counted the number of differentially expressed genes (DEGs) and the number of all annotated genes (All in brackets). Spine represents the upper jaw skin

**Fig. 6:**
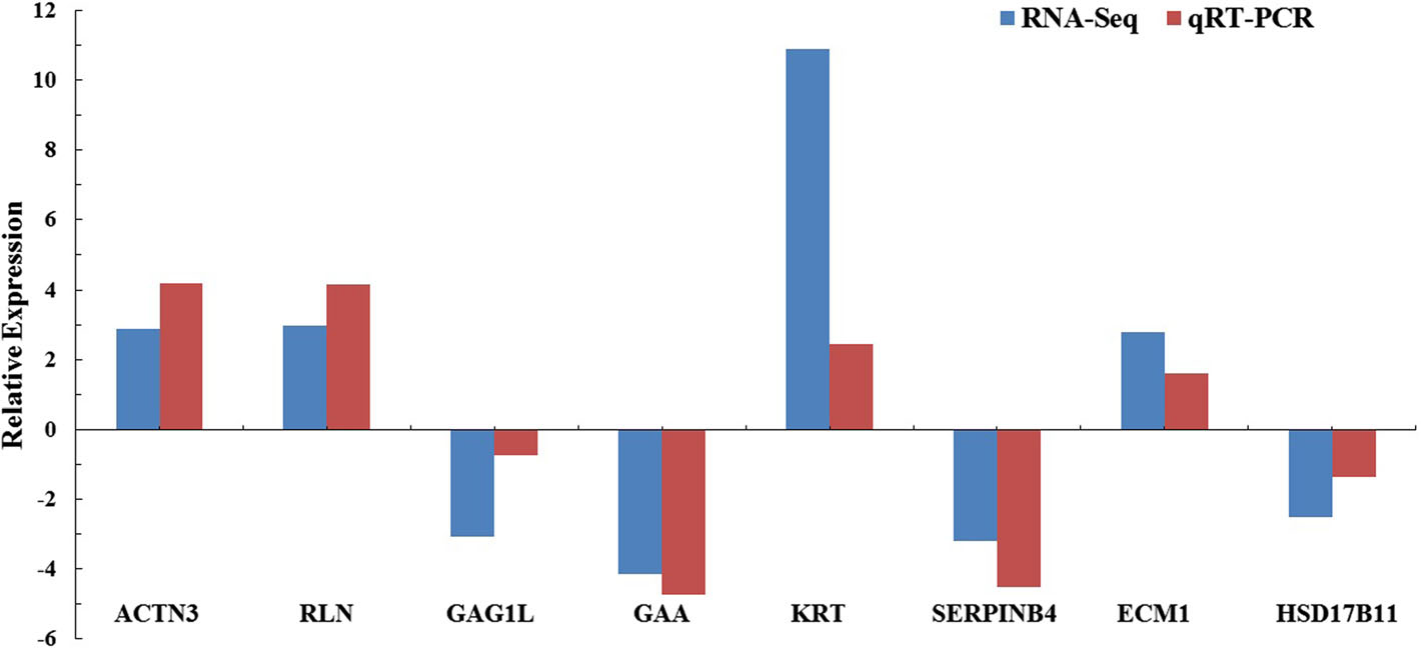
Quantitative real-time PCR confirmation of differentially expressed genes identified by RNA-seq. Relative expression levels are fold-changes expressed as the ratio of gene expression at the breeding period to post-breeding period normalized with GADPH gene

To validate the differentially expression profiles obtained from RNA-seq, we selected eight DEGs among the three tissues for qRT-PCR analysis. Six of them closely matched the results detected by RNA-seq, with a correlation coefficient of *R* = 0.957 (Fig. 6). Although the other two genes (GAG1L, KRT) did not have a perfect match and exhibited approximately 4-fold differences in relative expression, they still exhibited the same expression pattern at the two time periods. The correlation coefficient for all eight genes was *R =* 0.745. Overall, the qRT-PCR results were consistent with the RNA-sequencing data, indicating that our transcriptome data were credible.

#### Differentially expressed genes in the upper jaw skin

A total of 1,181 DEGs were identified in the upper jaw skin between the breeding and post-breeding periods in *L. boringii*. Among them, 748 genes (63.34%) were down-regulated, and the remaining 433 genes (36.66%) were up-regulated during the breeding period (Fig. 5c). A complete list of the DEGs in the upper jaw skin is provided (Additional file 1: Table S3). To better understand the biological processes of the DEGs in the skin during the two time periods of the development cycle for nuptial spines, we assigned all DEGs to GO and KEGG databases and analyzed the significant enrichments. However, despite the fact that so many DEGs were identified from the upper jaw skin, no significant enrichment of GO terms was identified among all DEGs (Additional file 1: Table S2). In up-regulated DEGs in the upper jaw skin, ncRNA metabolic process, tRNA metabolic process and ether hydrolase activity were found to be significantly enriched (adjust *P* < 0.05), indicating that the nuptial spines were actively transcribed during the growth period (Additional file 1: Table S2). The most enriched GO terms among DEGs (*P* < 0.05) in the skin were related to the cytosolic part, peptidase inhibitor activity, peptidase regulator activity and the extracellular region (Table 3a). This finding indicates that the seasonal development of the nuptial spines may be caused by changes in the cytosol cells and their extracellular matrix from the ‘dermis base’ of spines in upper jaw skin. The differential expression of peptidase inhibitor and regulator activities may be involved in the regulation of growth metabolism for the nuptial spines. The significantly enriched KEGG pathway among all DEGs in the upper jaw skin was only glycolysis/gluconeogenesis (Table 4). In addition, both the up-regulated and down-regulated genes were significantly enriched in the pathways related to the category of metabolism and genetic information processing. Yet, different biological processes were enriched between up-regulated and down-regulated genes (Table 4). During the breeding period, DEGs involved in translation and protein processing exhibited high expression levels, whereas during the post-breeding period, DEGs were mainly involved in the metabolism of cofactors and vitamins, amino acids and carbohydrates.

We identified the top 10 up-regulated and 10 down-regulated genes in the upper jaw skin by the order of FDR correction to obtain potential genes that may directly control the seasonal development of the nuptial spines. Our results indicate that the most differentially expressed genes in the upper jaw skin were mainly expressed in the extracellular matrix and were involved in peptidase inhibitor activity, the regulation of transcription and metabolic processes including COL28A1, SERPINB4, OGN and CSTB (Table 5). The top three most down-regulated genes during the breeding period were all lysosomal alpha-gluacosidase-like genes (Table 5). A previous study proposed that lysosome-mediated autophagy may participate in the regulation of cell death by degrading the extracellular matrix [40]. We thus considered that the epidermal cells of the upper jaw skin connected to the keratinized spines may undergo apoptosis when these spines are sloughed. Newt-specific cysteine-rich growth regulator (nsCCN) is a growth factor that was first identified in the newt species *Notophthalmus viridescens* and has an important role in the regeneration process of heart tissue [41]. In *L. boringii*, we observed a high expression level of nsCCN in the upper jaw skin after the spines had sloughed. Hence, we hypothesized that nsCCN may play a crucial role in the regeneration of epidermal cells after spines separate from the skin. The surface of the nuptial spines is highly keratinized, and the deciduous part is mainly the keratinized region. Interestingly, many of the keratin-related genes were up-regulated in the upper jaw skin when these spines developed. These genes were down-regulated when these weapons were sloughed (Additional file 1: Table S3). These findings support the involvement of keratin-related genes in the development of nuptial spines.

**Table 5:** Top 20 most differentially expressed genes in the upper jaw skin

Gene_Id	Symbol	FDR	log2FC	Expression	Gene Description
comp80990_c0	GAA	7.69E-21	−4.1233	Down	Lysosomal alpha-glucosidase-like
comp65666_c0	GAA	4.74E-20	−4.1481	Down	Lysosomal alpha-glucosidase-like
comp41689_c0	GAA	4.87E-18	−4.5083	Down	Lysosomal alpha-glucosidase-like
comp42769_c0	N/A	8.91E-16	−5.0603	Down	Hypothetical protein LOC100026915
comp78052_c0	COL28A1	4.91E-15	−3.6949	Down	Collagen alpha-1(XXVIII) chain
comp83419_c0	SERPINB4	1.54E-12	−3.196	Down	Serpin peptidase inhibitor, clade B (ovalbumin), member 4
comp95579_c0	OGN	2.13E-12	−3.0357	Down	Mimecan-like
comp82704_c0	N/A	7.28E-12	−4.6938	Down	UDP-GlcNAc: beta Gal beta-1,3-acetylglucosaminyl transferase 6 isoform 1
comp94451_c0	N/A	3.84E-11	−3.916	Down	Hypothetical protein LOC100493539
comp93492_c0	nsCCN	9.4E-11	−3.3559	Down	Newt-specific cysteine-rich growth regulator
comp87325_c0	N/A	3.55E-31	5.7757	Up	WAP four-disulfide core domain protein 3-like
comp70946_c0	CSTB	7.89E-23	4.2488	UP	Cystatin B (stefin B)
comp28513_c0	KRT42	1.93E-11	7.2399	Up	Keratin, type I cytoskeletal 42-like
comp95706_c0	CEACAM2	7.64E-10	2.7041	Up	Carcinoembryonic antigen-related Cell adhesion molecule 2-like, partial
comp90443_c0	HIGD1A	2.68E-09	2.6328	UP	HIG1 domain family, member 1A
comp94262_c0	CD59	3.23E-09	2.5861	Up	CD59 glycoprotein-like
comp75816_c0	TMEM229B	1.41E-08	2.548	Up	Transmembrane protein 229b-like
comp82708_c0	NPPC	3.42E-08	2.7999	Up	C-type natriuretic peptide 2
comp84554_c0	LDHA	3.87E-08	2.6708	Up	Lactate dehydrogenase A
comp20263_c0	N/A	5.16E-08	5.0778	Up	Hypothetical protein M91_20043

The gene ID, FDR, log2 FC, and gene description of the top 10 down-regulated and 10 up-regulated significantly differentially expressed genes (DEGs) are presented. Expression means gene expression level at the breeding period compared with the expression level at the post-breeding period (up- or down-regulated). We sorted the DEGs first by the smallest FDR and then by the largest absolute value of log2 FC. FDR, false discovery rate adjusted *P*-value. Log2 FC, log2 transformed fold-change values

#### Differentially expressed genes in the testis and brain

Using the same method as for the upper jaw skin, we further analyzed DEGs in the testis and brain. In summary, fewer DEGs were identified in these two tissues compared with the upper jaw skin. A total of 843 and 107 genes were differentially expressed in the testis and brain, respectively. In the testis, 499 genes (59.19%) were down-regulated and 344 genes (40.81%) were up-regulated during the breeding period (Fig. 5b; Additional file 1: Table S4). In the brain, the corresponding data were 52 genes (48.60%) and 55 genes (51.40%), respectively (Fig. 5a; Additional file 1: Table S5).

Among the three studied tissues, we found the greatest elevated number of enriched GO terms in the testis with 16 terms significantly enriched among all DEGs and 103 GO terms significantly enriched among down-regulated genes. The enriched GO terms were predominantly related to chromosome, DNA replication metabolism, cell division, cell cycle and spermatogenesis (Table 3b, Additional file 1: Table S2). Consistent with the GO term enrichment results, significant pathways enriched in KEGG were mostly assigned to genetic information processing, cellular processes, and metabolism (Table 4). Cell cycle, DNA replication, Cell cycle-yeast, and Meiosis-yeast were the most enriched pathways among all DEGs. The top most up-regulated and down-regulated genes identified in the testis were mainly related to the above function categories (Additional file 1: Table S6). Overall, the enrichment results overwhelmingly highlighted the activity of genetic processing and cellular processes in the testis. Generally, the seasonal expression of secondary sexual traits and sex accessory structures in most amphibians, especially the nuptial excrescences of anurans, are regulated by gonadal activity and gonadal steroid hormones [16]. However, we identified no significantly enriched terms related to steroid hormones in the testis. In *L. boringii*, time spent on parental care for offspring by reproducing males represents a significant proportion (greater than 70%) of the breeding season [32, 42]. Such a parental behavior is often correlated with low androgen levels in vertebrate males [21, 43]. Hence, the steroid hormone-related genes were rarely differentially expressed in the testis during the breeding season of *L. boringii*. We still find some DEGs in the testis that might be indirectly involved in steroid hormone activity. The relaxin and progesterone receptor genes are good examples of such genes (Additional file 1: Table S4). Therefore, we hypothesized that the gonadal activity may not directly participate in the regulation of the seasonal development of the nuptial spines through testosterone but though other factors such as estrogen-related genes.

Probably due to the limited number of DEGs in the brain, no significant enrichment of GO terms and KEGG pathways was identified. Among the enriched GO terms (*p* < 0.05), “aralkylamine N-acetyltransferase activity” was the most enriched term (Table 3c). Aralkylamine N-acetyltransferase is involved in the production of melatonin; it functions in the modulation of circadian rhythm and is essential for seasonal reproduction of vertebrates [44]. The top DEGs in the brain were mainly related to signal transduction (Additional file 1: Table S6). Among the top DEGs, kisspeptin precursor gene was the most down-regulated during the breeding period (Table 3). Kisspeptin is a G-protein coupled receptor ligand that can stimulate the secretion of aldosterone and the release of insulin [45]. Furthermore, we found a consistent expression pattern of ncCCN in the brain as well as in the upper jaw skin with ncCCN exhibiting high expression levels during the post-breeding period. Previous studies reported that the phenotypic variation of sexual traits would lead to alterations of gene expression in the brain [46]. Thus, we proposed that the brain may indirectly modulate the seasonal expression of nuptial spines through the genes specified above but also through other still unidentified genes.

### Potential genes regulating the seasonal development of the nuptial spines

Previous studies have proposed that the development and maintenance of male sexually selected traits in vertebrates often rely on the regulation of androgens, including testosterone [16, 21, 47]. In addition, the expression of SSTs tends to be more sensitive to the body condition than to other phenotypic traits of the holders, and recent discoveries implicated the widely conserved insulin/insulin-like growth factor pathway as the common physiological mechanism regulating the growth of these sexual traits [23, 24]. Based on our results and results of previous studies, we further selected DEGs with functional categories related to insulin/insulin-like growth factor and sex steroid hormone-related genes from all DEGs between the two studied time periods to identify potential genes regulating the seasonal development of the nuptial spines.

#### Insulin/insulin-like growth factor-related genes

As a result, we selected three significant DEGs between the two studied periods associated with insulin/insulin-like growth factor (IGFBP2, IGF2-B, nsCCN) in the upper jaw skin. Consistent with previous studies, IGFBP2 and IGF2-B revealed up-regulated expression when the nuptial spines were developed (Table 6). The insulin-like growth factors (IGF-I and IGF-II) are produced by most tissues of vertebrates and play roles in the regulation of proliferation and differentiation of cells in most tissues [26]. IGF-binding proteins (IGFBP) alter the interaction of IGFs with their cell surface receptors and modulate the function of the IGFs in cell culture [48]. The up-regulated IGF2-B and IGFBP2 genes during the breeding period may increase the metabolism and growth of the cells of the upper jaw skin connected with the nuptial spines, allowing the growth of these weapons. However, the exact molecular pathway involved must be verified by further experiments. Additionally, the up-regulated nsCCN in the upper jaw skin and brain when nuptial spines are sloughed may play an important role given their vital role in the tissue regeneration process.

**Table 6:** Significantly differentially expressed genes involved in the two candidate pathways in *Leptobrachium boringii*

Gene name	Gene Id	Tissues	Expression	|log2FC|	Description
Insulin/insulin-like growth factor-related genes					
IGFBP2	comp91565_c0	Spine	Up	1.8375	Insulin-like growth factor-binding protein 2 precursor
IGF2-B	comp95271_c0	Spine	Up	1.4543	Insulin-like growth factor II-B precursor
nsCCN	comp93492_c0	Spine	Down	3.3566	Newt-specific cysteine-rich growth regulator
nsCCN	comp93492_c0	Brain	Down	2.3587	Newt-specific cysteine-rich growth regulator
Steroid hormone-related genes					
HSD17B11	comp93113_c2	Spine	Down	2.5100	Hydroxysteroid (17-beta) dehydrogenase 11
HSD17B6	comp93072_c0	Spine	Down	2.2184	Hydroxysteroid (17-beta) dehydrogenase 6 homolog precursor
HSD17B2	comp82544_c0	Spine	Down	1.7356	Estradiol 17-beta-dehydrogenase 2-like
ESR1	comp77282_c0	Spine	Down	1.6833	Estrogen receptor alpha
fRLX	comp81782_c0	Testis	Up	2.9616	Relaxin
PGR	comp92661_c0	Testis	Up	1.8223	Progesterone receptor

Expression indicates gene expression level at the breeding period compared with the expression level at the post-breeding period (up- or down-regulated). |log2FC|, the absolute value of log2 transformed fold-change. Spine represents the upper jaw skin

The expression of striking sexual traits is always associated with the holder’s body condition, as their expression is commonly sensitive to the nutrition history [24, 49]. However, prior studies on condition-dependent sexual traits rarely focused on amphibians, and such sexual traits are often tightly coupled with individual variation within a population [25]. In our study, for the first time, we found that the expression of a striking male sexual trait was likely associated with the changes in physiological condition during a reproductive cycle of an amphibian species. Insulin-like growth factor 2 (IGF2) may be the main peptide affecting the development of nuptial spines in *L. boringii*, and this is different from the regulation pattern for exaggerated traits in most vertebrates through IGF1 [23, 27]. In conclusion, the seasonal development of the nuptial spines in *L. boringii* may operate in a condition-dependent manner, and the IGF2 gene may be involved in the regulation of this development.

#### Sex steroid hormone-related genes

Five significantly DEGs involved in sex steroid hormone-related pathways were identified in the upper jaw skin, and two related DEGs were identified in the testis (Table 6). Seasonally breeding amphibians often undergo dramatic changes in the concentration of sex hormones and in reproductive characteristics such as sexual morphological traits and reproductive behaviors triggered by those hormones at the onset and termination of each breeding cycle [16, 50]. Specifically, 17β-hydroxysteroid dehydrogenases (17β-HSD) are a group of alcohol oxidoreductases that play a key role in the regulation of intracellular concentrations of steroid hormones, including androgens and estrogens [51, 52]. CYP3A5 encodes a member of the cytochrome P450 enzyme superfamily catalyzing the metabolism of steroid hormone into less biologically active metabolites [53, 54]. During the post-breeding period, the high expression of 17β-HSD genes (HSD17B2, HSD17B6 and HSD17B11) and CYP3A5 may increase the degradation and metabolism of steroid hormones and thus decrease the concentration of active androgen and estrogen in the upper jaw skin. Generally, testosterone regulates the expression of male secondary sexual traits directly by stimulating intracellular androgen receptors in the target tissues or indirectly by aromatizing androgens into estrogen and binding to estrogen receptors [21]. Interestingly, in the upper jaw skin, no androgen receptor but an estrogen receptor gene (NR3A1) was differentially expressed between the breeding and the post-breeding periods (Table 6). Once activated, NR3A1 (or ERα) can translocate into the nucleus and regulate the activity of eukaryotic gene expression, cellular proliferation and differentiation in target tissues [55, 56]. However, to date, only a few amphibian species have been reported to have α isoform of the estrogen receptor (ER) [57, 58]. Our study is the first to report the expression of ERα in the skin of an amphibian species, indicating that the upper jaw skin where the spines develop may be a target tissue whose growth and replacement are regulated by estrogen.

Commonly, the presence of male parental care behavior will decrease the expression of secondary sexual traits [28]. However, male *L. boringii* express and maintain nuptial spines, a secondary sexual trait, during the entire breeding season while simultaneously taking care of offspring for a relatively long time period (at least two months) [31, 42]. Together with the low androgen-dependent parental care behavior in *L. boringii*, the seasonal acquisition and maintenance of nuptial spines may be regulated by estrogen. Moreover, the up-regulated 7β-HSD genes and ERα would have an inhibitory effect on androgen output during the post-breeding period [59, 60]. Thus, estrogens may play an important role in the seasonal regulation of the nuptial spines in *L. boringii*. However, elucidating whether the seasonal development of the nuptial spines is regulated by androgen directly, by aromatization of androgen into estrogens exerting related effects, or by both still needs further investigation.

Relaxin is a small peptide hormone that can enhance male sperm motility and fertilization capacity in vertebrates [61, 62]. The expression of fRLX was altered with the annual reproductive cycle of a frog species *Pelophylax esculentus* and regulated by testosterone [63, 64]. The up-regulated fRLX in the testis of *L. boringii* during the breeding period may enhance male reproductive activity. The progesterone receptor (PGR) gene was also up-regulated in the testis during the breeding period. This gene is activated by progesterone and can inhibit androgen-dependent reproductive behaviors in males [65, 66]. Thus, we hypothesize that the high expression of PGR during the breeding season may increase the propensity of males for providing parental care at the expense of courtship and mating activities. Finally, there may be other unknown important genes participating in the regulation of seasonal expression for this trait in *L. boringii*; thus, further study is needed.

Taken together, candidate genes involved in insulin/insulin-like growth factor and sex steroid hormone pathways indicate that the nuptial spines in *L. boringii* exhibit high estrogen sensitivity and low insulin/insulin-like growth factor sensitivity during the post-breeding period when the spines are sloughed. Thus, the seasonal development and maintenance of the nuptial spines in male *L. boringii* are likely modulated by the circulating sex steroid hormones and the nutritional status. Candidate genes in the testis and brain may indirectly participate in this process by regulating the synthesis and metabolism activity of steroid hormones and insulin growth factor. Further study should focus on these two candidate pathways and use testable experiments to validate the molecular mechanism.

## Conclusions

In this study, we conducted a transcriptome analysis of an anuran species to explore the genetic basis of the seasonal growth of a sexual weapon trait. Our overall transcriptome provided a rich list of unigenes expressed in adult males of *L. boringii* during the breeding and the post-breeding periods. DEGs between different growth periods of the keratinized nuptial spines in *L. boringii* were identified. In total, 94,900 unigenes and 2,131 DEGs were generated in our transcriptome. These genes greatly contribute to current genetic resources for the anuran species. The number of DEGs identified from the upper jaw skin was larger than the testis and the brain, indicating that the upper jaw skin might contribute more to the seasonal changes in the spine activity. Candidate genes related to steroid hormone signaling pathways and the insulin/insulin-like growth factor pathway are likely involved in regulating the seasonal growth of the spines. The candidate genes and pathways are promising for future studies on the seasonal growth of the nuptial spines in *Leptobrachium* species and may be helpful for other vertebrates.

## Methods

### Sample collection and RNA extraction

*L. boringii* male individuals were caught from the population of the Badagong Mountain Natural Reserve (Hunan, China, 29°39′-29°49′ N, 109°41′-110°09′ E) during the breeding season of 2014. Sampling was performed during the breeding period (in late March when the nuptial spines were developed) and during the subsequent post-breeding period (in late May once the spines had sloughed, Fig. 1). At each sampling time, three individuals were caught as biological replicates for each study period. Once caught, these toads were kept in single plastic boxes containing the same plant leaves and water from their natural environment and transported to the laboratory. The living toads were euthanized by injecting buffered MS-222 solution (3 g/l) through the dorsal skin. Each toad was dissected with 0.1% diethyl pyrocarbonate (DEPC)-ddH_2_O solution-treated scissors, and a tissue sample (each approximately 100 mg in scale) was taken from the brain, testis and the upper jaw skin and stored in liquid nitrogen until needed. Specifically, the tissue upper jaw skin was taken from the skin area that contained a ‘papillary dermis base’ of the spine, and the tissue brain was taken from the whole brain region of each sampled individual. The testis sample was randomly taken from the whole tissue with the certain portion of each individual.

Total RNA of each sample was extracted using an RNA extraction kit (Omega Bio-Tek, USA) with TRIzol® Reagent (Invitrogen, CA, USA) following the manufacturer’s instructions. RNA degradation and contamination was tested using 1% agarose gels. We measured RNA purity and RNA concentration using the NanoPhotometer® spectrophotometer (IMPLEN, CA, USA) and Qubit® RNA Assay Kit in Qubit® 2.0 Flurometer (Life Technologies, CA, USA), respectively. RNA integrity was assessed using the RNA Nano 6000 Assay Kit of the Agilent Bioanalyzer 2100 system (Agilent Technologies, CA, USA). Total RNA was extracted from each tissue sample separately for three replicates of each breeding period, and 18 high-quality RNA samples were finally acquired.

### Illumina sequencing and *de novo* assembly

For each tissue, using 3 μg total RNA as input material, a cDNA library was constructed using the NEBNext® Ultra™ RNA Library Prep Kit for Illumina® (NEB, USA) following the manufacturer’s instructions. In brief, mRNA was purified from total RNA using poly-T oligo (dT) magnetic beads, and fragments were generated using divalent cations under elevated temperature in NEBNext First Strand Synthesis Reaction Buffer (5×). First strand cDNA was synthesized using random hexamer primers and M-MuLV Reverse Transcriptase (RNase H^−^), and second strand cDNA was subsequently synthesized with DNA Polymerase I and RNase H. The library fragments with a preferential length of 150 to 200 bp were purified with the AMPure XP system (Beckman Coulter, Beverly, USA). Then, PCR was performed with Phusion High-Fidelity DNA polymerase, universal PCR primers and Index (X) Primer. We purified PCR products using the AMPure XP system and assessed library quality using the Agilent Bioanalyzer 2100 system. The above cDNA samples were then clustered using the TruSeq PE Cluster Kit (Illumina, USA) on a cBot Cluster Generation System. We sequenced all libraries on an Illumina Hiseq 2000 platform (San Diego, CA, USA), and 100 bp paired-end reads were then generated for each of them.

Clean reads were obtained by removing the trimming adapter sequences, reads containing poly-N and low quality reads (reads with greater than 5% unknown nucleotides or reads with less than 13 bp) from raw reads. The number of raw reads, clean reads and Q20 were calculated at the same time. All subsequent analyses were based on the clean reads. Due to the absence of reference sequences for *L. boringii*, high quality clean reads were *de novo* assembled using Trinity software (r20140717) with the min_kmer_cov set to 2 and with default values for all remaining parameters [67]. Unigenes was selected from the longest transcript copy of each gene clusters to avoid redundant transcripts [68]. All unigenes from the 18 tissue samples were combined in a unigene database for the studied species, and all subsequent analyses were performed on this database.

### Functional annotation of unigenes

To functionally annotate the unigenes in *L. boringii*, we searched our unigene set against the datasets of non-redundant protein sequences (NR) in the National Center for Biotechnology Information (NCBI; http://www.ncbi.nlm.nih.gov), the Protein family database (PFAM; http://pfam.xfam.org/) and the manually annotated and reviewed protein sequence database (Swiss-Prot database; http://www.ebi.ac.uk/swissprot/) using NCBI-BLAST (v 2.2.30) [69] with an *E*-value cut-off of 1 × 10^−5^. Annotation was extended to the Functional classifications of Gene Ontology (GO; http://www.geneontology.org/), the Clusters of Orthologous Groups of proteins (KOG/COG; http://www.ncbi.nlm.nih.gov/COG), and Pathway Annotation of the Kyoto Encyclopedia of Genes and Genomes (KEGG; http://www.genome.jp/kegg/pathway.html). GO annotation was performed using Blast2GO pipelines with an *E*-value ≤ 1E-3 [70, 71]. KOG annotation was searched with an *E*-value ≤1E-5 [38]. We searched the unigenes against the KEGG database using KOBAS (v 2.0) [72].

### Differential expression analyses

Gene expression levels in each tissue were estimated using the software package RSEM (v 1.2.0) based on the expectation-maximization (EM) algorithm [73]. Firstly, clean reads of each sample were mapped back onto the assembled transcripts. Then, the read count for each gene was obtained from the mapping results. The gene expression level was estimated by calculating the fragments per kb per million reads (FPKM) [74]. The profiles of gene expression level generated for each tissue were combined and used to detect DEGs between the breeding and the post-breeding periods. We used DESeq R package (v2.15.3) [75] to perform differential gene expression analyses in the three tissues between these two time periods. The resulting *p*-values were adjusted using the Benjamini and Hochberg’s approach for controlling the false discovery rate [76]. Genes with an adjusted *p-*value (FDR) < 0.05 and |log2 (fold-change)| > 1 were assigned as significant level.

### Enrichment analysis of differentially expressed genes

GO enrichment of the DEGs was analyzed using GOseq R packages based on Wallenius non-central hyper-geometric distribution [77], which can adjust for gene length bias in DEGs. The smallest *P*-value indicates the highest degree of GO enrichment. KEGG pathway enrichment of the DEGs was tested using KOBAS software [72]. The hypergeometric test was applied in the enrichment analysis. The minimum threshold of statistical significance was fixed at *p* = 0.05.

### Data confirmation by quantitative PCR analysis

To confirm the accuracy of our transcriptome data, we selected eight genes among the significantly DEGs in the three tissues and performed quantitative PCR experiments. PCRs were performed with a CFX96 Touch Real-Time PCR Detection System (BIO-RAD, Hercules, CA, USA) using SYBR Green I Dye with a 20-μl total volume mixture containing 2 μl of cDNA as the template. The expression levels of the tested genes were normalized with a housekeeping gene (glyceraldehyde-3-phosphate dehydrogenase, GAPDH) as an internal control, and relative gene expression was analyzed with the 2^-△△Ct^ method [78]. Mean values and standard errors were determined by three biological replicates. The specific primers for these genes were designed using PRIMER 5, and information on these primers is additionally provided (Additional file 1: Table S7).

## Additional files

Additional file 1: Table S1. The statistics and quality of reads throughout all samples in the *Leptobrachium boringii* transcriptome*.* **Table S2.** Significant enrichment of GO terms in the three tissues. **Table S3.** Differentially expressed genes in the upper jaw skin. **Table S4.** Differentially expressed genes in the testis. **Table S5.** Differentially expressed genes in the brain. **Table S6.** Top 10 most differentially expressed genes in testis and brain of *Leptobrachium boringii*. **Table S7.** Specific primers used for quantitative PCR validation. (XLSX 769 kb)
Additional file 2: Figure S1. Heatmap comparing differentially expressed genes of the three tissues between different breeding periods. (DOCX 265 kb)

## Abbreviations

CYP3A5: Cytochrome P450 Family 3 subfamily a member 5
DEG: Differentially expressed genes
FDR: False discovery rate adjust *p* value
GO: Gene ontology
HSD17B: 17-beta-hydroxysteroid dehydrogenase
IGF: Insulin/insulin-like growth factor
IGFBP: Insulin-like growth factor-binding protein
KEGG: Kyoto encyclopedia of genes and genomes
KOG/COG: Clusters of orthologous groups of proteins
NR: Non-redundant protein sequences
NR3A1: Estrogen receptor 1
nsCCN: Newt-specific cysteine-rich growth regulator
PGR: Progesterone receptor
PFAM: Protein family database
qRT-PCR: Quantitative reverse transcription real-time PCR
SSTs: Sexually selected traits
Swiss-Prot: The manually annotated and reviewed protein sequence database.
