# Supplementary material for: Transcriptome analysis reveals the genetic basis underlying the seasonal development of keratinized nuptial spines in Leptobrachium boringii
Source: BMC Genomics. 2016 Nov 28;17:978. doi: 10.1186/s12864-016-3295-9 (PMC5126826; doi:10.1186/s12864-016-3295-9)
Supplement: Additional file 2: Figure S1. — Heatmap comparing differentially expressed genes of the three tissues between different breeding periods. (DOCX 265 kb) [file 12864_2016_3295_MOESM2_ESM.docx]

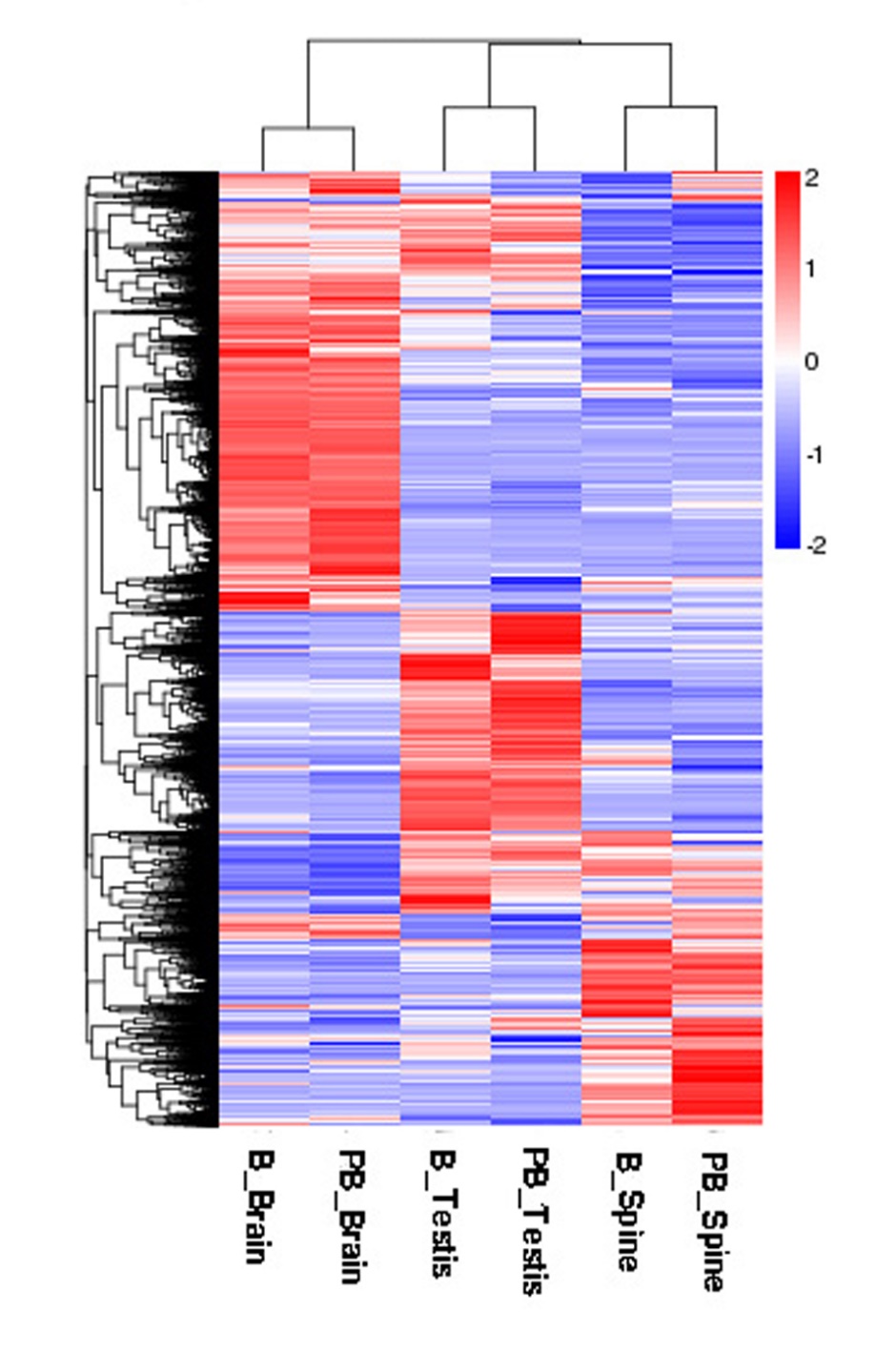


Figure S1 Heatmap comparing differentially expressed genes at the three tissues between different breeding periods. The intensity of colors indicates expression levels of the genes. B and PB represented the breeding period and post breeding period, respectively. Spine represented the upper jaw skin for short.
